# Features and associated factors of bacterial skin infections in hospitalized patients with pemphigus: a single-center retrospective study

**DOI:** 10.1186/s12941-020-00388-6

**Published:** 2020-10-08

**Authors:** Furong Li, Yejun Wu, Wenjie Bian, Lei Huang, Xuejun Zhu, Xixue Chen, Mingyue Wang

**Affiliations:** 1 Department of Dermatology, Peking University First Hospital, 8 Xishiku Street, Xicheng District, Beijing, 100034 China; 2 National Clinical Research Center for Skin and Immune Diseases, Beijing, China; 3 Beijing Key Laboratory of Molecular Diagnosis on Dermatoses, Beijing, China; 4 Department of Eight-year Clinical Medical Education, Peking University People’s Hospital, Beijing, China; 5 Department of Clinical Laboratory, Peking University First Hospital, Beijing, China

**Keywords:** Bacterial skin infections, Pemphigus, Antibiotics, Gram’s stain, *Staphylococcus aureus*

## Abstract

**Background:**

Infections were the primary cause of death (34.3–55.5%) in patients with pemphigus. Skin was usually the origin of infections. The study aimed to explore features and associated factors of bacterial skin infections (BSIs) in inpatients with pemphigus.

**Methods:**

One hundred and seventy-seven inpatients with pemphigus hospitalizing from November 2014 to April 2019 were continuously recruited through Peking University First Hospital’s inpatient records inpatients with pemphigus hospitalizing from November 2014 to April 2019 were continuously recruited through Peking University First Hospital’s inpatient records. Then, we retrieved the clinical and laboratory data to explore the characteristics and associated factors of BSIs.

**Results:**

Of patients enrolled, pemphigus vulgaris (PV, n = 142) and pemphigus foliaceus (PF, n = 9) were most common, followed by pemphigus erythematosus (PE, n = 25) and pemphigus vegetans (Pveg, n = 1). Eighty-seven of 177 (49.2%) inpatients developed BSIs, and they had a longer length of stay compared with inpatients without BSIs (median: 18.9 vs. 14.1 days, p = 0.008). *Staphylococcus aureus* was the most common bacteria (71.3%, 62/87) and highly resistant to penicillin (91.9%, 57/62). Higher levels of anti-Dsg1 autoantibodies (> 124.2 U/mL) (p < 0.001, odds ratio [OR] = 3.564, 95% confidence interval [CI]: 1.784–7.123) and anti-Dsg3 autoantibodies (> 169.5 U/mL) (p = 0.03, OR = 2.074, 95% CI: 1.084–3.969) were underlying risk factors of BSIs when analyzed by binary regression analysis. As for Gram’s stain of bacteria, females had a lower rate of Gram-positive infections (p = 0.03). Patients using oral antibiotics (p = 0.05) had a higher rate of Gram-negative infections. Inpatients who were hospitalized in other hospitals within 2 weeks before the current admission had a higher rate of Gram-negative and co-infections (p = 0.03).

**Conclusions:**

Inpatients with pemphigus had a high incidence of BSIs. Some factors were associated with the susceptibility of BSIs and bacterial species.

## Introduction

Pemphigus is an autoimmune bullous disease characterized by flaccid blisters and erosions of skin/mucous membranes [1]. Annual incidence varies from 0.76 to 16.1 cases per million [2, 3]. Subtypes of pemphigus were differentiated by clinical features, histopathology, and specific autoantibodies against desmogleins (Dsg) [2]. Pemphigus vulgaris (PV) (65%) and pemphigus foliaceus (PF) (23%) are the most frequent subtypes [4]. Though the use of glucocorticoids and immunosuppressive agents has significantly diminished the mortality, the prognosis of pemphigus is still worse than that of the general population [2]. Infections are the most frequent complications of patients with pemphigus and account for 34.3–55.5% of all death [5–8]. Besides, inpatients with pemphigus have a higher rate of serious infections than inpatients without a diagnosis of autoimmune bullous disease (50.4 vs. 25.4%) [9]. In pemphigus with serious infections, hospital expenses increase, and length of stay (LOS) extends [9]. Skin is usually the origin of infections [10]. Fragile barrier, dysfunction of immunity, and usage of systemic corticosteroids and/or other immunosuppressing agents may be the causes [5–8]. Therefore, the study aims to explore the features and associated factors for bacterial skin infections (BSIs), which may be helpful for the control of BSIs.

## Materials and methods

### Patients and data collection

This was a retrospective study conducted at Peking University First Hospital starting in May 2019. The study followed the Declaration of Helsinki and was approved by the ethics committee of the hospital. One hundred and seventy-seven inpatients with pemphigus were continuously recruited from November 2014 to April 2019. Diagnostic criteria of pemphigus included: (1) flaccid blisters and erosions on skin or mucosa, (2) suprabasal/subcorneal loss of epidermal adhesion in histopathology, (3) IgG and/or C3 deposits on the surface of keratinocytes, and (4) seropositivity of anti-Dsg1 and/or anti-Dsg3 autoantibodies (MBL, Nagoya, Japan) [11]. Bacterial skin infections (BSIs) were diagnosed by purulent skin secretions, fever, elevated inflammatory markers (white blood cells, neutrophils, C-reactive protein, etc.), and culture of skin swabs. A culture of skin swab was necessary for the diagnosis of BSIs. If the isolated bacteria might be contaminating bacterium, clinical features and other laboratory examinations would be thoroughly considered for the diagnosis of BSIs. BSIs identified within the first 48 h was identified as community-acquired infections [12]. The final diagnosis of BSIs was made by a consultant doctor in infectious diseases and a senior dermatologist. Body mass index (BMI) more than 28 kg/m^2^ was defined as obesity [13]. Hypoalbuminemia was defined as < 30 g/L [14].

Demographic characteristics, history of previous hospitalization, the severity of skin lesions, etc. were retrieved from the hospital inpatient databases. We mainly pulled levels of anti-Dsg antibodies and albumin, white blood cell count, C-reactive protein, culture results, and antibiotic susceptibility test results out of the laboratory database. The treatment protocols within 2 weeks before this hospitalization were also collected. The severity of skin lesions graded as follows: mild, < 10.0% body surface area (BSA); moderate, 10.0–30.0% BSA; severe, 30.0–50.0% BSA; extensive, ≥ 50.0% BSA. The systemic glucocorticoids was graded as follows (equivalent to prednisone): low dosage: < 0.5 mg/(kg·day); medium dosage: ≥ 0.5 mg/(kg·day) and < 1.0 mg/(kg·day); high dosage ≥ 1.0 mg/(kg·day).

### Bacterial identification and antimicrobial susceptibility testing

The specimens were streaked into both Columbia blood agar plates and China Blue agar plates at 35 °C for 24–48 h until single colonies were obviously seen. Then the single colony was sub-cultured when necessary. The pure colonies from the primary culture or sub-culture were selected for further bacterial identification.

Bacterial identification was performed by Bruker Daltonik MALDI Biotyper system (BD, USA), with MALDI Biotyper 3.1 software. For the bacteria with identification scores lower than 1.699, PCR amplification and DNA sequencing of 16S rRNA genes were performed as described previously [15].

Antimicrobial susceptibility testing was performed by VITEK-2 Compact automated system (BioMerieux, France) following the manufacturer’s instructions, with AST-P639 card for *Staphylococcus aureus (S. aureus )* isolates included in our study. The determined minimal inhibitory concentrations (MICs) were interpreted as susceptible, intermediate, or resistant according to the breakpoints from Clinical and Laboratory Standards Institutes M100-S29 document, *Staphylococcus aureus* (*S. aureus*) ATCC 25923 was used as quality control in each set of tests [16].

### Statistical analysis

**Fig. 1:**
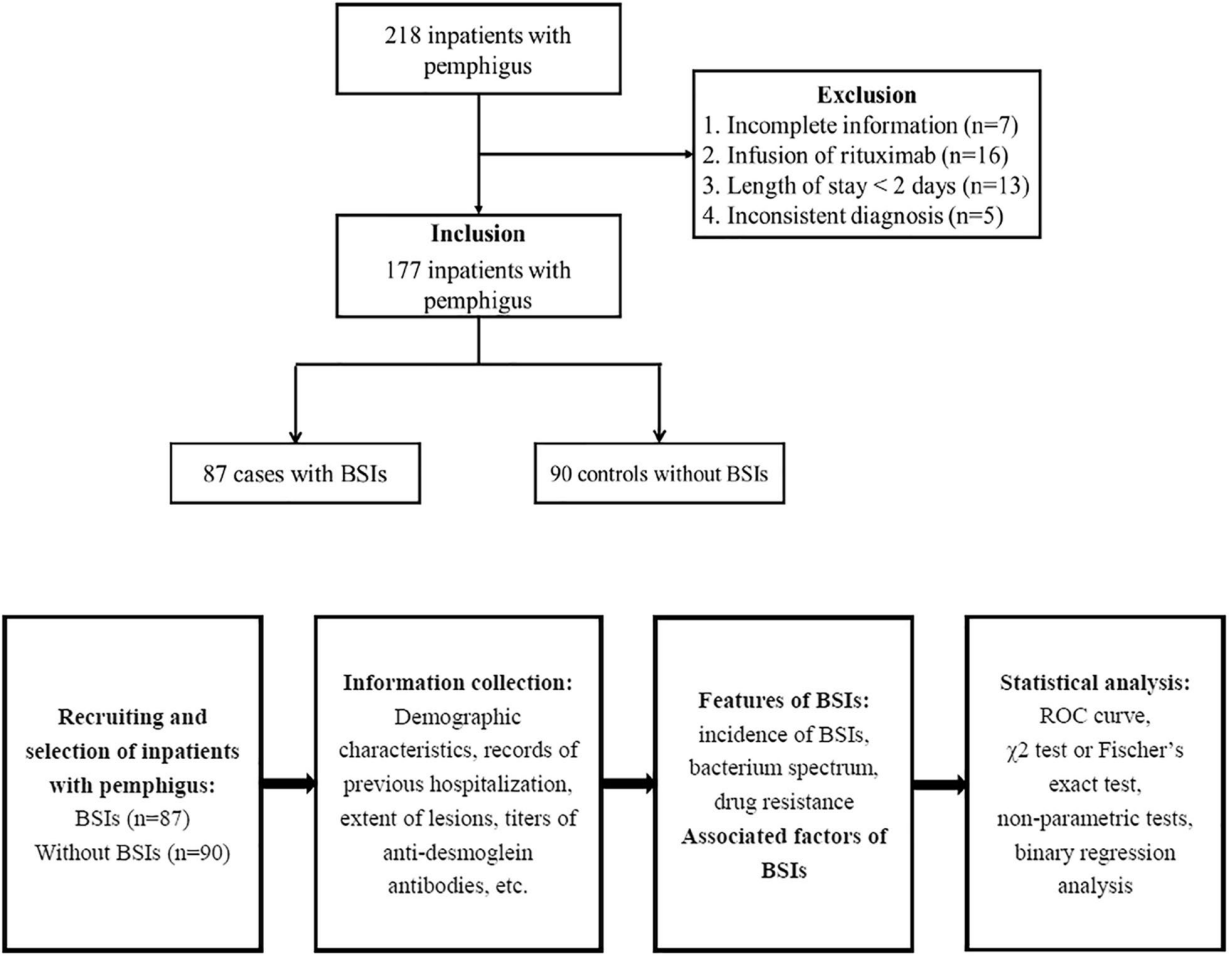
Flow diagram of the study

Data were analyzed with IBM SPSS 24.0 (IBM Corp, Armonk, NY). Continuous variables were converted into categorical variables by the Receiver Operating Characteristics curve, by which anti-Dsg1 and anti-Dsg3 autoantibodies were grouped by 124.2 and 169.5 IU/ml, respectively, and age was grouped by 53.5 years. Non-parametric tests were used for variables not meeting the normal distribution. Chi-square test or Fisher’s exact test were used for group variables. Factors with P ≤ 0.10 were reanalyzed by binary regression analysis. P < 0.05 was considered statistically significant. See Fig. 1 for the study and analysis flow chart.

## Results

### Clinical features

A total number of 177 inpatients were enrolled, including PV (80.2%, 142/177), pemphigus erythematosus (PE, a subtype of PF) (14.1%, 25/177), PF (5.1%, 9/177), and pemphigus vegetans (Pveg, a subtype of PV) (0.6%, 1/177). The median age was 50.4 years (range: 14–80 years), and the female/male ratio was 0.90. The incidence of diabetes mellitus (DM) (18.6%, 33/177), hypertension (27.7%, 49 /177), and osteoporosis (11.3%, 20/184) were high. Twelve (6.8%), 31 (17.5%), 52 (29.4%) and 82 (46.3%) of 177 patients had mild, moderate, severe and extensive skin lesions, respectively. Most inpatients (70.6%, 125/177) received oral glucocorticoids, and 114 (91.2%) of 125 had detailed records of the dosage. Of them, 43.9% (50/114) patients took medium dose (0.5–1.0 mg/kg·day), and 24.6% (28/114) patients took high dose (≥ 1.0 mg/kg·day) of prednisone or the equivalent. Eighty-seven of 177 (49.2%) inpatients developed BSIs. LOS was analyzed by a non-parametric test, and inpatients with BSIs had longer LOS compared with inpatients without BSIs (median: 18.9 vs. 14.1 days, p = 0.008).

### Bacterial spectrum and drug resistance

**Fig. 2:**
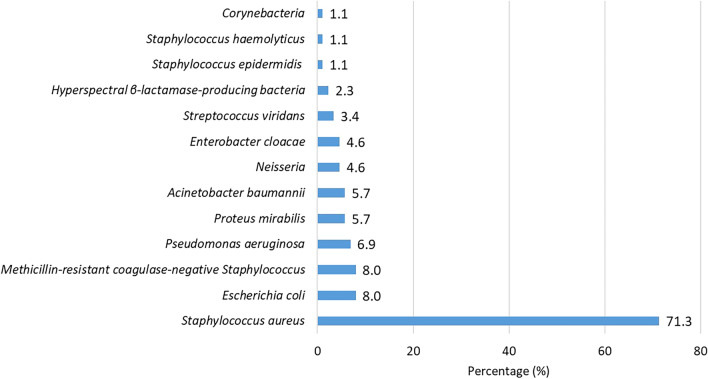
Bacteria spectrum of inpatients with pemphigus

**Fig. 3:**
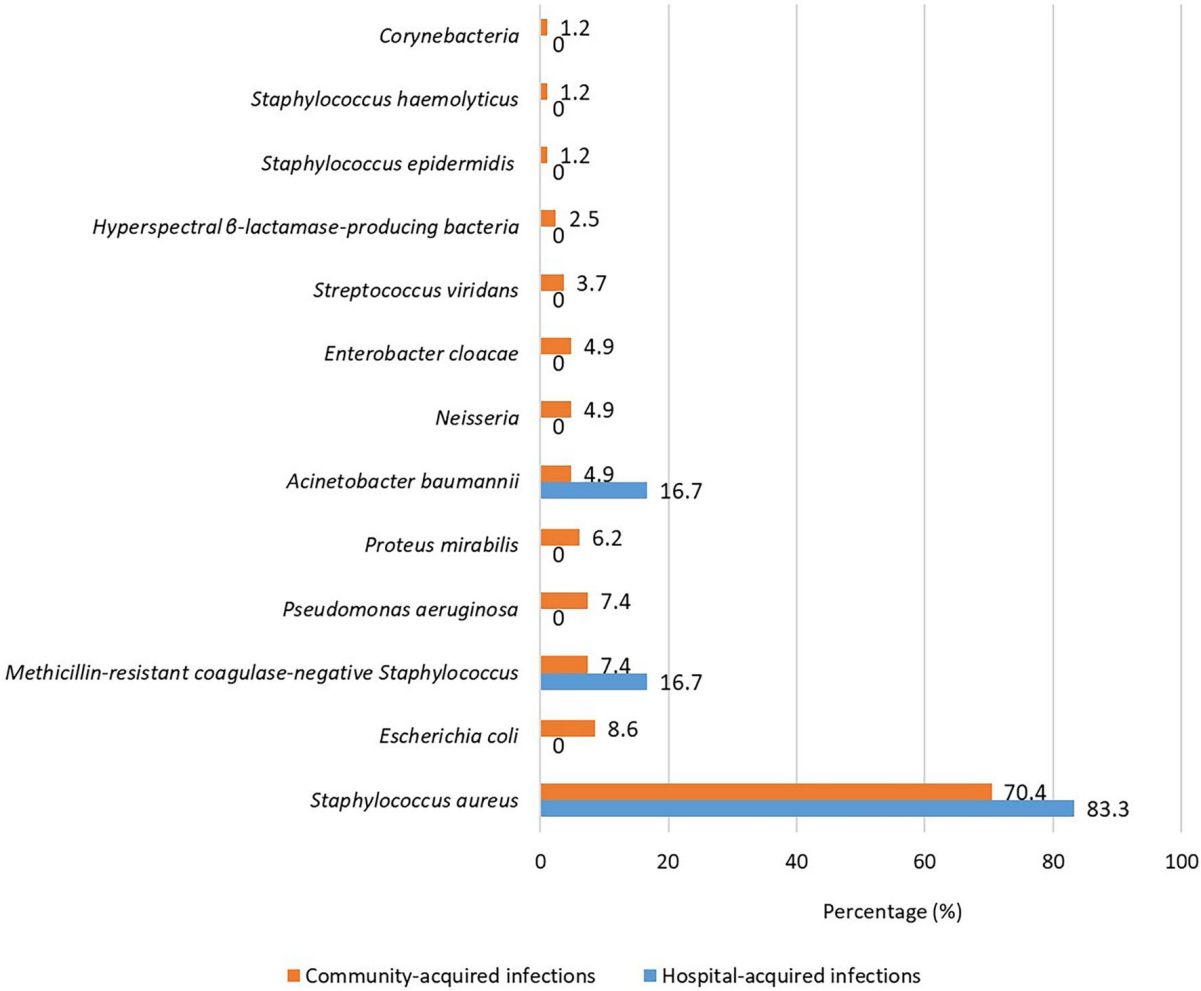
Bacteria spectrum of community-acquired infections and hospital-acquired infections

Eighty-seven of 177 (49.2%) patients developed BSIs, and 17 of 87 (19.5%) inpatients had multiple bacterial infections. If a patient, who was diagnosed with BSIs, had the isolations of both pathogenic bacteria and underlying contaminating bacteria, such as *Staphylococcus epidermidis* *or Streptococcus viridans*, it was hard to differentiate whether the *Staphylococcus epidermidis or Streptococcus viridans* was contaminating bacteria or not. Therefore, we showed all the isolated bacteria of these inpatients. The Bacterial spectrum of all inpatients with BSIs was presented in Fig. 2. Sixty-two (71.3%, 62/87) inpatients were infected with *S. aureus*, followed by *Escherichia coli* (8.0%, 7/87), and methicillin-resistant coagulase-negative *Staphylococcus* (8.0%, 7/87). Eighty-one (93.1%, 81/87) inpatients presented with community-acquired infections. Patients with hospital-acquired infections had a higher incidence of *S. aureus* (83.3%, 5/6). The bacterial spectrum of community-acquired infections and hospital-acquired infection infections was illustrated in Fig. 3.

It was a pity that many patients were not record the location of collected strains. Forty-three patients (43/87, 49.4%) detailed recorded the location of collected strains, including the scalp (13/43, 30.2%), the back (12/43, 27.9%), the axilla (12/43, 27.9%), and the other sites (33/43, 76.7%). The most common bacteria of the scalp, the back, and the axilla were *S.aureus* (7/13, 53.8%), *S.aureus* (8/12, 66.7%), and *Enterobacter cloacae* (3/12, 25.0%), respectively.

**Fig. 4:**
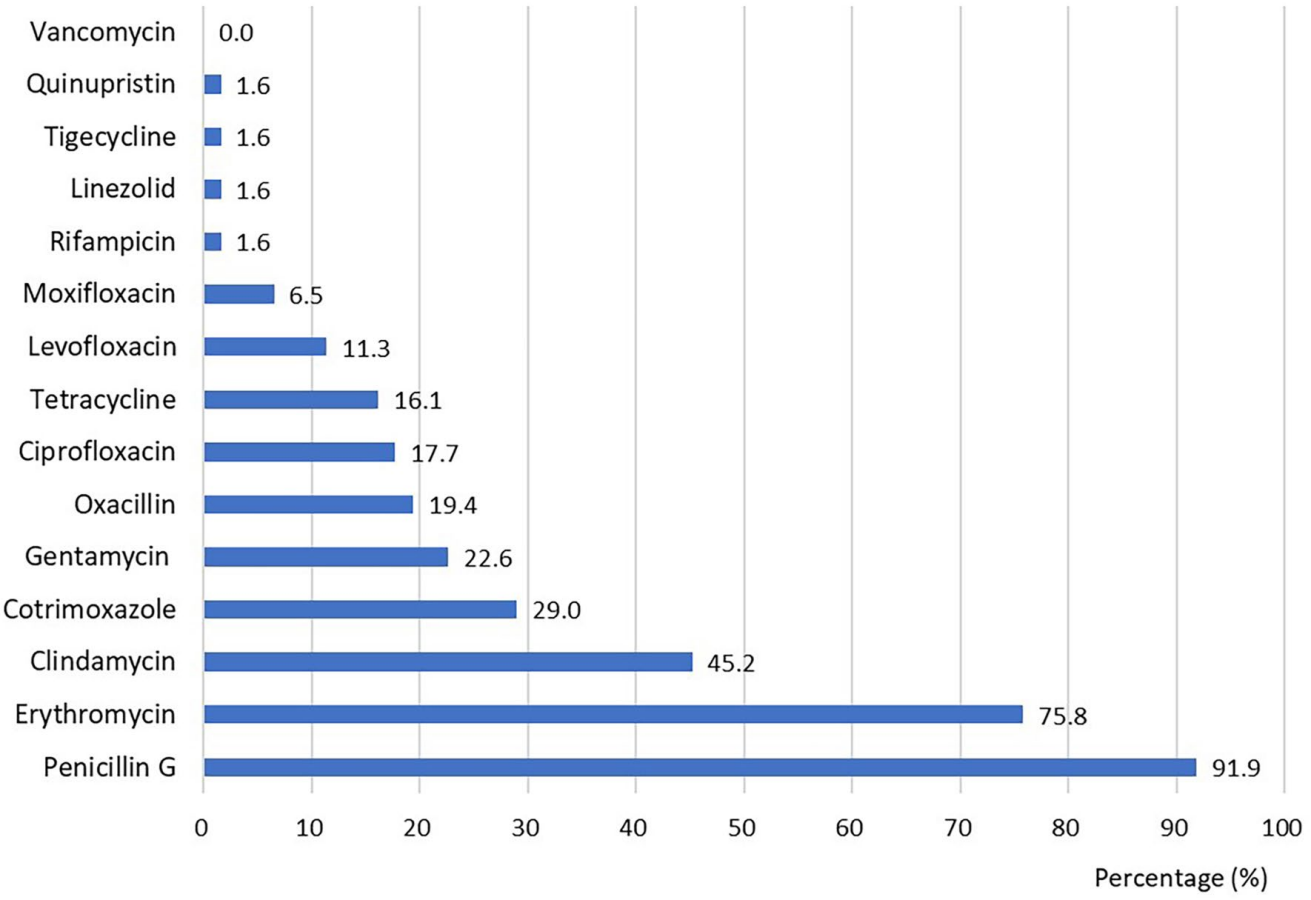
Drug resistance of *Staphylococcus aureus*

As for the drug resistance of *S. aureus*, penicillin G (91.9%, 57/62), erythromycin (75.8%, 47/62), and clindamycin (45.2%, 28/62) had the highest resistance rate. The resistance rate of rifampicin (1.6%, 1/62), linezolid (1.6%, 1/62), quinupristin (1.6%, 1/62), and tigecycline (1.6%, 1/62) was the lowest. All patients were sensitive to vancomycin (Fig. 4). As the second most common bacteria, methicillin-resistant coagulase-negative *Staphylococcus* was also highly resistant to penicillin (100.0%, 7/7) and erythromycin (100.0%, 7/7). Methicillin-resistant coagulase-negative *staphylococcus* remained 100% sensitive to tigecycline and vancomycin.

### Risk factors for BSIs

Sex (p = 0.49), age (p = 0.96), obesity (p = 0.24), and smoking (p = 0.13) did not significantly associate with BSIs. Cerebrovascular disease (CVD) (p = 0.96), chronic heart disease (CHD) (p = 0.34), diabetes mellitus (DM) (p = 0.28), hypertension (p = 0.72), and osteoporosis (p = 0.58) did not associate with higher incidence of BSIs. Similarly, severity of skin lesions (p = 0.08), subtype of pemphigus (p = 0.51), and hypoalbuminemia (p = 0.09) were not underlying risk factors for BSIs. Higher levels of anti-Dsg1 autoantibodies (> 124.2 U/mL) (p < 0.001) and anti-Dsg3 autoantibodies (> 169.5 U/mL) (p = 0.01) were associated with BSIs (Table 1). The variables with p ≤ 0.10 were reanalyzed by binary regression analysis and the results showed in Table 2. Higher levels of anti-Dsg1 autoantibodies (> 124.2 U/mL) (p < 0.001, odds ratio [OR] = 3.564, 95% CI: 1.784–7.123) and anti-Dsg3 autoantibodies (> 169.5 U/mL) (p = 0.03, OR = 2.074, 95% CI: 1.084–3.969) were significantly associated with BSIs.

**Table 1:** Associated factors of BSIs by univariate analysis

Features	Without BSIs (n = 90)	BSIs (n = 87)	*P*-value
Sex			0.49
Female (n = 84)	45 (50.0)	39 (44.8)	
Male (n = 93)	45 (50.0)	48 (55.2)	
Age			0.96
< 53.5 years (n = 100)	51 (56.7)	49 (56.3)	
≥ 53.5 years (n = 77)	39 (43.3)	38 (43.7)	
Obesity (n = 25)^a^	10 (11.4)	15 (17.6)	0.24
Smoking (n = 16)	11 (12.2)	5 (5.7)	0.13
Alcohol intake (n = 11)	8 (8.9)	3 (3.4)	0.13
Comorbidities			
CVD (n = 7)	3 (3.3)	4 (4.6)	0.96
CHD (n = 5)	1 (1.1)	4 (4.6)	0.34
Diabetes mellitus (n = 33)	14 (15.6)	19 (21.8)	0.28
Hypertension (n = 49)	26 (28.9)	23(26.4)	0.72
Osteoporosis (n = 20)	9 (10.0)	11 (12.6)	0.58
Bedridden (n = 2)	0 (0.0)	2 (2.3)	0.24
Previous hospitalization (n = 18)	10 (11.1)	8 (9.2)	0.67
The severity of skin lesions			*0.08*
Mild (n = 12)	9 (10.0)	3 (3.4)	
Moderate (n = 31)	20 (22.2)	11 (12.6)	
Severe (n = 52)	24 (26.7)	28 (32.2)	
Extensive (n = 82)	37 (41.1)	45 (51.7)	
Subtype of pemphigus			0.51
PV and PVeg (n = 143)	71 (78.9)	72 (82.8)	
PE and PF (n = 34)	19 (21.1)	15 (17.2)	
Therapy			
Antibiotics (n = 40)	20 (22.2)	20 (23.0)	0.90
Glucocorticoids (n = 129)	65 (72.2)	64 (73.6)	0.84
Immunosuppressive agent (n = 43)	26 (28.9)	17 (19.5)	0.15
Hypoalbuminemia (n = 21)	7 (8.0)	14 (16.3)	*0.09*
Anti-Dsg1 autoantibodies^b^			< *0.001*
≤ 124.2 IU/ml (n = 61)	44 (50.6)	17 (20.5)	
> 124.2 IU/ml (n = 109)	43 (49.4)	66 (79.5)	
Anti-Dsg3 autoantibodies^c^			*0.01*
≤ 169.5 IU/ml (n = 93)	56 (64.4)	37 (44.6)	
> 169.5 IU/ml (n = 77)	31 (35.6)	46 (55.4)	

^a^Four patients (without BSIs = 2, BSIs = 2) had no records of BMI so that these patients could not be assessed for obesity

^b^Seven patients had no records of levels of anti-dsg1 autoantibodies (without BSIs = 3, BSIs = 4)

^c^Seven patients had no records of levels of anti-dsg3 autoantibodies (without BSIs = 3, BSIs = 4)

CVD, cerebrovascular disease; CHD, coronary heart disease; PV, Pemphigus vulgaris; PVeg, pemphigus vegetans; PE, pemphigus erythematosus; PF, pemphigus foliaceus; Dsg, desmoglein; BSIs, bacterial skin infections; BMI, body mass index

**Table 2:** Associated factors of BSIs by binary regression analysis

Variables	OR (95% CI)	*P*-value
Anti-Dsg1 autoantibodies		< *0.001*
≤ 124.2 IU/mL	1.00	
> 124.2 IU/mL	3.564 (1.784–7.123)	
Anti-Dsg3 autoantibodies		*0.03*
≤ 169.5	1.00	
> 169.5	2.074 (1.084–3.969)	

BSIs, bacterial skin infections; Dsg, desmoglein; OR, odds ratio; CI, confidence interval

### Associated factors for the type of bacteria

In inpatients with BSIs, 60 (69.0%, 60/87), 13 (14.9%, 13/87), and 14 (16.1%, 14/87) had infections of Gram-positive bacteria, Gram-negative bacteria and coinfections, respectively. Females had a higher rate of coinfection (54.3 vs. 35.7%) and a lower rate of Gram-negative infection (25.4 vs. 84.6%) compared to males (p = 0.03). Though the result was not significantly different, a higher rate of coinfections was observed in patients <53.5 years compared to ≥ 53.5 years’ (71.4 vs. 28.6%) (p = 0.37). Comorbidities, including CVD (p = 0.72), CHD (p = 0.72), DM (p = 0.39), hypertension (p = 0.86), and osteoporosis (p = 0.92), were not associated with Gram’s stain. Bedridden was not significantly associated with Gram’s stain (p = 0.09). Within 2 weeks before this admission, patients taking oral antibiotics had a higher rate of Gram-negative isolation (p = 0.05). Patients who were hospitalized in other hospitals within two weeks before the current admission had higher rates of Gram-negative infection and co-infections (p = 0.03) (Table 3).

**Table 3:** Associated factors of Gram’s stain of bacteria in patients with pemphigus by univariate analysis

Features	Gram-positive (n = 60, %)	Gram-negative (n = 13, %)	Coinfection (n = 14, %)	*P*-value
Sex				*0.03*
Female (n = 39)	28 (46.7)^a,b^	2 (15.4)^b^	9 (64.3)^a^	
Male (n = 48)	35 (54.7)^a,b^	11 (84.6)^b^	5 (35.7)^a^	
Age				
< 53.5 years (n = 49)	31 (51.7)	8 (61.5)	10 (71.4)	0.37
≥ 53.5 years (n = 38)	29 (48.3)	5 (38.5)	4 (28.6)	
Obesity (n = 15) ^1^	8 (13.3)	5 (41.7)	2 (15.4)	0.10
Smoking (n = 5)	5 (8.3)	0 (0.0)	0 (0.0)	0.15
Alcohol intake (n = 3)	2 (3.3)	1 (7.7)	0 (0.0)	0.47
Comorbidities				
CVD (n = 4)	2 (3.3)	1 (7.7)	1 (7.1)	0.72
CHD (n = 4)	2 (3.3)	1 (7.7)	1 (7.1)	0.72
Diabetes mellitus (n = 19)	11 (18.3)	3 (23.1)	5 (35.7)	0.39
Hypertension (n = 23)	16 (26.7)	4 (30.8)	3 (21.4)	0.86
Osteoporosis (n = 11)	7 (11.7)	2 (15.4)	2 (14.3)	0.92
Bedridden (n = 2)	0 (0.0)	1 (7.7)	1 (7.1)	*0.09*
Previous hospitalization (n = 8)	2 (3.3)^a^	3 (23.1)^b^	3 (21.4)^b^	*0.03*
The severity of skin lesions				0.40
Mild (n = 3)	1 (1.7)	1 (7.7)	1 (7.1)	
Moderate (n = 11)	7 (11.7)	3 (23.1)	1 (7.1)	
Severe (n = 28)	17 (28.3)	4 (30.8)	7 (50.0)	
Extensive (n = 45)	35 (58.3)	5 (38.5)	5 (35.7)	
Subtype of pemphigus				0.92
PV and PVeg (n = 72)	49 (81.7)	11 (84.6)	12 (85.7)	
PE and PF (n = 15)	11 (18.3)	2 (15.4)	2 (14.3)	
Therapy				
Topical antibiotics (n = 10)	5 (8.3)	3 (23.1)	2 (14.3)	0.35
Systemic antibiotics (n = 11)	4 (6.7)^a^	4 (30.8)^b^	3 (21.4)^a,b^	*0.05*
Topical glucocorticoids (n = 7)	6 (10.0)	1 (7.7)	0 (0.0)	0.27
Systemic glucocorticoids (n = 62)	40 (66.7)	10 (76.9)	12 (85.7)	0.29
Immunosuppressive agents (n = 17)	13 (21.7)	2 (15.4)	2 (14.3)	0.75
Hypoalbuminemia (n = 14)	8 (13.6)	3 (23.1)	3 (21.4)	0.61
Anti-Dsg1autoantibodies^2^				0.29
≤ 124.2 IU/mL (n = 17)	9 (16.1)	3 (23.1)	5 (35.7)	
> 124.2 IU/mL (n = 66)	47 (83.9)	10 (76.9)	9 (64.3)	
Anti-Dsg3 autoantibodies^3^				0.77
≤ 169.5 IU/mL (n = 37)	26 (46.4)	6 (46.2)	5 (35.7)	
> 169.5 IU/mL (n = 46)	30 (53.6)	7 (53.8)	9 (64.3)	

^1^ Two patients (Gram-positive = 0, Gram-negative = 1, Coinfection = 1) had no records of body mass index so that these patients could not be assessed for obesity

^2^ Four patients had no records of levels of anti-dsg1 autoantibodies (Gram-positive = 4, Gram-negative = 0, Coinfection = 0)

^3^ Four patients had no records of levels of anti-dsg3 autoantibodies (Gram-positive = 4, Gram-negative = 0, Coinfection = 0)

CVD, cerebrovascular disease; CHD, coronary heart disease; PV, *Pemphigus vulgaris*; PVeg, pemphigus vegetans; PE, pemphigus erythematosus; PF, pemphigus foliaceus; Dsg, desmoglein

## Discussion/conclusion

Infection was the most common comorbidity in patients with pemphigus [9, 17], and increased hospital expenses and LOS [9]. Our study also found the LOS prolonged in inpatients with BSIs (p = 0.008). Ninety-four (49.2%, 87/177) inpatients with pemphigus developed BSIs in our research, parallel with another study (52%, 73/141) [18]. *S. aureus* was the most common type of bacteria [10, 19–21]. The drug resistance of *S. aureus* was striking [19]. *S. aureus* was highly resistant to penicillin (91.9%), erythromycin (75.8%), and clindamycin (45.2%). *S. aureus* was sensitive to vancomycin and moxifloxacin, which might be good choices for inpatients who need empirical and vigorous treatment.

Associated factors for BSIs were explored. Hsu et al. suggested that patients who had multi-morbidities and poor health conditions were more likely to develop lethal infections [17], and DM was associated with infections in many studies [1, 18, 19, 22]. However, comorbidities, such as DM, CVD, CHD, etc., were not significantly associated with BSIs in our study. Hypoalbuminemia was suggested to be associated with poor health conditions and had a higher rate of BSIs in previous studies [23, 24], but no association was observed in our analysis.

Many studies linked infections to immunosuppressive agents [25, 26]. Hsu et al. proposed that infection in patients with pemphigus was iatrogenic. The role of glucocorticoids and other immunosuppressive agents of infection was uncertain [17]. Glucocorticoids and immunosuppressive agents were not significantly associated with BSIs in our studies. It might be due to the influences of both immunosuppression and promoting recovery of skin lesions of these drugs. Although 82 of 177 (46.3%) patients had extensive skin lesions, only 43 of 177 (24.3%) patients accepted immunosuppressors before his hospitalization. The nonstandard treatment also influenced the association between immunosuppressors and BSIs.

Higher levels of anti-Dsg1 autoantibodies (> 124.2 U/mL) (p < 0.001) and anti-Dsg3 autoantibodies (> 169.5 U/mL) (p = 0.03) were significantly associated with BSIs. Patients with high levels of anti-Dsg1 autoantibodies or anti-Dsg3 autoantibodies had high disease activity and severe or even recalcitrant skin lesions [27–29], which could explain the phenomena. However, the association between the area of skin lesions and BSIs was not significant (p = 0.08) in our study, which might be because we only briefly calculated the area of skin lesions and did not consider the types of skin damage for the incomplete record.

We also paid attention to the associated factors of the type of bacteria. Female had higher incidence of coinfection (64.3 vs. 35.7%), and lower incidence of Gram-negative infection (15.4 vs.84.6%) (p = 0.03) compared to males in our study. Different gender-related lifestyle and physical function might cause this difference [30–32]. The discrepancy of body-size, immune capacity, and energy availability between males and females might be the underlying reasons. Besides, Thompson et al. found that pathogen transmission and virulence were much higher in females [33]. Anyway, the underlying interesting mechanism of different infections in different gender was unclear and needed further studies. The use of systemic antibiotics was associated with a higher rate of Gram-negative infections. Dysbacteriosis induced by antibiotics might be the reason [34]. Patients who were hospitalized in other hospitals within two weeks before the current admission (p = 0.03) had a higher rate of Gram-negative infection and co-infections. It was known that the bacteria spectrum of the hospital was different from the community. For example, the most common bacteria of community-acquired pneumonia was *Streptococcus pneumonia,* while the most common bacteria of hospital-acquired pneumonia was *Acinetobacter baumannii* and *Pseudomonas aeruginosa* [35, 36]. Iatrogenic manipulation and the use of antibiotics caused the difference of bacterial spectrum [37, 38]. So we should pay more attention to the infections of Gram-negative bacteria in inpatients with a history of previous hospitalization.

## Limitations

The study has some limitations. This was a retrospective study in a single center, so the generalizability might be influenced, and selection bias existed. This study did not enroll outpatients with pemphigus, which might influence the spectrum of bacteria and drug resistance. The relatively small sample size was also a disadvantage of this study. Patients admitted to the tertiary hospital might be more serious, so the incidence of BSIs might be overestimated. Finally, the authors did not exclude the underlying contaminating bacteria in showing the species of isolated bacteria. Though the limitations exist, the results proposed in the study are beneficial to understand the BSIs and may be the basis for high-quality researches in the future.

## Conclusions

The study found that inpatients with pemphigus had a high incidence rate of BSIs, and *S. aureus* was the most common isolated bacteria. High levels of anti-Dsg1 (> 124.2 U/mL) and Dsg3 autoantibodies (> 169.5 U/mL) might be underlying risk factors for BSIs. Besides, gender, systemic usage of antibiotics, and history of the previous hospitalization might influence the type of isolated bacteria. We highlighted the importance of further exploration of underlying risk factors of BSIs in inpatients with pemphigus.

## Data Availability

The datasets of the current study are available from the corresponding author on reasonable request.

## Abbreviations

AIBD: Autoimmune bullous disease
BMI: Body mass index
BSA: Body surface area
BSIs: Bacterial skin infections
CHD: Chronic heart disease
CI: Confidence interval
CVD: Cerebrovascular disease
DM: Diabetes mellitus
Dsg: Desmoglein
LOS: Length of stay
OR: Odds ratio
PE: Pemphigus erythematosus
PF: Pemphigus foliaceus
PV: Pemphigus vulgaris
Pveg: Pemphigus vegetans
